# The Moderating Role of Anxiety and Depressive Symptoms in Protective Effects of Health Behaviors among Clients Using Mental Health Services

**DOI:** 10.3390/jcm13061753

**Published:** 2024-03-18

**Authors:** Yusen Zhai, Mahmood Almaawali, Xue Du

**Affiliations:** 1 Department of Human Studies, The University of Alabama at Birmingham, Birmingham, AL 35294, USA; 2 Psychology Department, Sultan Qaboos University, Al-Khod 123, Oman; mawali89@squ.edu.om; 3 Heersink School of Medicine, The University of Alabama at Birmingham, Birmingham, AL 35294, USA; dux@uab.edu

**Keywords:** anxiety, depression, health behaviors, infectious disease, clients, mental health services

## Abstract

College-student clients using mental health services contend with increased anxiety and depressive symptoms, and their vulnerability to infectious respiratory diseases and severe clinical outcomes rises. To mitigate severe outcomes, health behaviors serve as essential protective tools to reduce the risk of infectious diseases, including COVID-19. Considering the escalating prevalence of anxiety and depression among college-student clients, little is known about how anxiety and depressive symptoms could potentially attenuate the protective effects of COVID-19 health behaviors (i.e., masking, social distancing, and hygiene practice). This study aims to examine the interactive effects of anxiety/depression and health behaviors in predicting COVID-19 infection. **Methods**: We analyzed data from the 2020–2021 Healthy Mind Study including a random sample of 9884 college-student clients in mental health services across 140 higher education institutions in the United States. We performed multivariable logistic regression to assess whether and to what extent the associations between COVID-19 health behaviors and infection depended on severity of anxiety or depressive symptoms. **Results**: Anxiety symptom severity negatively moderated the protective effects of social distancing against infection after adjusting for demographic characteristics and pre-existing chronic health conditions. Depressive symptom severity negatively moderated the protective effects of masking, social distancing, or hygiene practices against infection. **Conclusion**: The associations between certain COVID-19 health behaviors and infection were conditional on anxiety and depressive symptom severity. Findings suggest a potential public health benefit of mental health clinicians’ efforts in assessing and treating clients’ anxiety and depressive symptoms, namely reducing their vulnerability to COVID-19 infection and perhaps other infectious respiratory diseases.

## 1. Introduction

Respiratory diseases contribute to economic burdens and severe health outcomes, such as hospitalizations and deaths. Lessons learned from the pandemic may help inform public health policy and research on other infectious respiratory illnesses. Coronavirus disease 2019 (COVID-19), caused by severe acute respiratory syndrome coronavirus 2 (SARS-CoV-2), has been responsible for approximately 1.18 million deaths in the United States as of February 2024 [1]. Between September 5th, 2020, and May 29th, 2021, the total number of hospitalizations and deaths reached 2 million and 0.4 million, respectively [1]. During this period, the average weekly hospitalizations and deaths surpassed 54,458 and 10,571, respectively [1], despite over 146 million people being fully vaccinated by May 29th, 2021 [2]. This period witnessed the first peak of COVID-19 infection in the U.S., specifically during January of 2021 [1]. While the national emergency status for COVID-19 was lifted in April 2023, the situation has not yet stabilized. From April 15th, 2023, to February 3rd, 2024, weekly new COVID-19 hospitalizations have seen a 60.2% increase from 13,280 to 21,271, and the weekly number of new COVID-19 deaths has escalated by 29.4% from 1218 to 1576 [1]. According to the U.S. CDC [3], COVID-19 persists as one of the leading causes of death. This alarming rise, in the backdrop of relaxed restrictions, calls for ongoing research to understand the impact of COVID-19 on health outcomes across various subpopulations, particularly those at a higher risk of infection [4]. The COVID-19 mortality rate for people with mental health conditions is two to three times higher than that of the general population [5,6]. Their increased vulnerability to infectious diseases and severe clinical outcomes is likely due to impaired cognition, risk behaviors, and altered immune function, including a pro-inflammatory state and maladaptive T-cell functioning, further exacerbated by sleep issues, loneliness, and social isolation [4,6,7,8,9,10], which requires essential protective measures, such as health behaviors, to ameliorate their risk of infection [5,6,7]. Research has shown that college students grappled with pressing mental health concerns during the pandemic [11,12]. There has been a growing body of literature documenting the relationships between mental health conditions (e.g., anxiety and depression) and the uptake of COVID-19 health behaviors, providing important public health insights [13,14]. However, the relationships between mental health conditions and the protective effects of these health behaviors remain understudied. Given the escalating prevalence of anxiety [64.9%] and depression [46%] among college-student clients receiving mental health services [15], a more granular understanding about the potential role of mental health symptom severity in the protective effects of health behaviors against COVID-19 infection can inform clinical practices and health policies to reduce the risk of severe health outcomes among this population. To bridge the research gap, we aimed to examine the interactive effects of anxiety/depression and health behaviors in predicting COVID-19 infection while controlling for demographic variables and plausible risk factors for COVID-19 infections to reduce possible confounding effects. This current study addressed the following research questions and the accompanying hypotheses:

**Research** **Question** **1.** *Does college-student clients’ severity of anxiety symptoms moderate the protective effects of their health behaviors (i.e., masking, social distancing, and hygiene practice) against COVID-19 infection?*

**Hypothesis** **1.** The associations between college-student clients’ COVID-19 health behaviors and infection would depend on their anxiety symptom severity.

**Research** **Question** **2.** *Does college-student clients’ severity of depressive symptoms moderate the protective effects of their health behaviors (i.e., masking, social distancing, and hygiene practice) against COVID-19 infection?*

**Hypothesis** **2:** The associations between college-student clients’ COVID-19 health behaviors and infection would depend on their depressive symptom severity.

## 2. Methods

### 2.1. Participants

The present study was approved by the institutional review board of the first author’s institution. Secondary, de-identified data was analyzed from the Healthy Minds Study (HMS), conducted from September 2020 to May 2021 across 140 U.S. higher education institutions and including 136,502 college students. The study, approved by IRB at each participating institution, required written informed consent from all participants. Diversity was present in the study sites regarding the institutional type, enrollment size, and geographic location (urban and rural campuses from all nine census regions). At institutions with over 4000 individuals, a random sample of 4000 was invited. Conversely, at smaller institutions, all individuals received an invitation.

The study adjusted differences between respondents and non-respondents through sample weights. These weights were based on institutional data about sex, race, academic level, and grade point average. Although the participants represented the entire population at each institution, certain demographic characteristics, such as sex and race, might differ between respondents and non-respondents. Institutional data was therefore utilized to calculate the response propensity for each participant type. Subsequently, each participant was assigned a response propensity weight, with larger weights given to those with underrepresented characteristics to ensure representation of the full institutional population.

In this present study, the population of interest was college-student clients using mental health services, given the high prevalence of anxiety and depression among this population. The inclusion criteria for this study stipulated that participants must be college students who were over the age of 18 and currently receiving mental health services; the exclusion criteria stipulated that individuals who were under the age of 18, not enrolled in a higher education institution, or not currently using mental health services, would be excluded from the study. Of the 136,502 students responding to the HMS survey, 9884 students responded “Yes” to a survey question (“Are you currently receiving counseling or therapy?”) while also meeting other inclusion criteria. Therefore, these 9884 participants who reported the current use of mental health services (e.g., university counseling centers or community providers) were included in this current study.

### 2.2. COVID-19 Infection

COVID-19 infection was measured by one question: “*Have you had COVID-19 (the novel coronavirus disease)*?” Participants answered this question based on their experience during the pandemic. This outcome was coded as a binary variable to indicate participants’ COVID-19 diagnosis/symptoms [16].

### 2.3. Severity of Anxiety Symptoms

The severity of anxiety symptoms was measured using the Generalized Anxiety Disorder–7 (GAD−7) [17]. The GAD−7 is a seven-item measurement used to screen for generalized anxiety disorder and measure anxiety symptom severity. This measurement uses a 4-point Likert scale ranging from 0, *Not at all*, to 3, *Nearly every day*. When evaluating anxiety symptom severity, the GAD−7 score ranges from 0 to 21; a higher score suggests more severe anxiety symptoms. Particularly, the cutoff of 10 (sensitivity, 0.89; specificity, 0.82) and 15 (sensitivity, 0.48; specificity, 0.95) might be considered as representing moderate and severe levels of anxiety [17]. The GAD−7 has consistently exhibited strong validity and reliability in studies conducted both before and during the pandemic [17,18]. It has sufficient construct, factorial, and procedural validity [19]. Furthermore, the GAD−7 showed good test–retest reliability over a 7-day period (r = 0.83) [19]. In the current sample, McDonald’s omega (ω) reliability of the GAD−7 was 0.90.

### 2.4. Severity of Depressive Symptoms

The severity of depressive symptoms was measured using the Patient Health Questionnaire–9 (PHQ−9) [20]. The PHQ−9 is a nine-item measurement used to screen depression and monitor depressive symptom severity. This measurement uses a 4-point Likert scale ranging from 0, *Not at all*, to 3, *Nearly every day*. When evaluating depressive symptom severity, the PHQ−9 score ranges from 0 to 27; a higher score suggests more severe depressive symptoms. Particularly, the cutoff of 10 (sensitivity, 0.88; specificity, 0.88) and 15 (sensitivity, 0.68; specificity, 0.95) might be considered as representing moderate and severe levels of depression [20]. The PHQ−9 has been consistently shown to have strong validity and reliability in studies conducted both before and during the pandemic [20,21]. Its construct and factorial validity have been well established [20,21]. In addition to the excellent test–retest reliability observed at 14 days (r = 0.94) [22], the PHQ−9 also exhibited high interrater reliability (ICC = 0.94) [23]. In the current sample, McDonald’s ω reliability of the PHQ−9 was 0.89.

### 2.5. COVID-19 Health Behaviors

COVID-19 health behaviors were measured using the three following questions: hygiene practices (“How closely have you been following recommendations for hygiene practices [frequent hand washing; avoiding touching your eyes, nose, and mouth; and disinfecting surfaces]?”), social distancing (“How closely have you been following recommendations for social/physical distancing [keeping a six-foot distance between yourself and others in public, avoiding gatherings of 10 or more people, and avoiding non-essential trips outside your home]?”), and masking (“How often do you wear a facemask in public when it is required?”). For questions about hygiene practices and social distancing, participants could respond from 1, *Not at all*, to 4, *Very closely*. For the question about masking, participants could respond from 1, *Never*, to 5, *All the time*. Higher scores refer to better adherence to health behaviors.

### 2.6. Covariates

Participants’ demographic variables, as covariates in our statistics models, included age (18–21, 22–25, 26–30, ≥31) [24], biological sex, race/ethnicity, disability status, socioeconomic status (i.e., financial situation), and geographic region. Because of known associations between pre-existing chronic health conditions and COVID-19 infection [25], our models controlled for these chronic health conditions to reduce potential confounding effects.

### 2.7. Data Analysis

We first performed logistic regression to compute adjusted odds ratios (aORs) and 95% confidence intervals (CIs) to examine the associations of the two predictors (i.e., anxiety/depressive symptom severity and health behavior) with COVID-19 infection, controlling for covariates, which allowed us to estimate the main effect of each health behavior on COVID-19 infection. As we aimed to test our two hypotheses by examining the interactive effects of anxiety/depression and health behaviors in predicting COVID-19 infection, we then added interaction terms into our models to determine whether the severity of anxiety or depressive symptoms would moderate the protective effects of health behaviors against COVID-19 infection. In stratified logistic regression models by the two predictors, the variables that represented the severity of anxiety or depressive symptoms were continuous variables, with a higher score indicating more severe symptoms. Likewise, the variables that represented health behaviors were continuous variables, with a higher score indicating better adherence.

As an abbreviated illustration of the modeling approach using a logistic regression framework including the variables previously described, the logistic model with an interaction can be written as follows:

$$\begin{array}{ccc} logitY_{i}=\beta_{0} & + & \beta_{1}{SymptomSeverity}_{i}+\;\beta_{2}{HealthBehavior}_{i}\\ & & +\;\beta_{3}{SymptomSeverity}_{i}\;*\;{HealthBehavior}_{i}\\ & & +\;\beta_{4}Covariates_{i}+\;\epsilon_{i} \end{array}$$

In this equation, *i* indexes participants. *Y_i_* refers to the study outcome of interest (i.e., COVID-19 infection status). *Covariates* indicate the demographic characteristics and chronic health conditions of participants. *ϵ* refers to the error term. *β*s are the estimated effects of one unit change in corresponding variables on the log odds of *Y*. The coefficient (i.e., *β*_3_) of the interaction term, as the primary variable of interest, is the estimator of the protective effect of one health behavior on the log odds of *Y* moderated by the severity of anxiety or depressive symptoms. A significant interaction term indicates that the severity of anxiety or depressive symptoms moderates the protective effect of a health behavior against COVID-19 infection. In each model, when the interaction term is statistically significant and positive, the negative association between the use of one health behavior and the odds of COVID-19 infection (as indicated by *β*_2,_ which was negative in all our models) becomes less negative as the severity of anxiety or depressive symptoms increases. Namely, a positive coefficient (i.e., *β*_3_) suggests that the protective effect of a health behavior against COVID-19 infection decreases as the severity of anxiety or depressive symptoms increases.

We assessed multicollinearity in our stratified models, and the results of the variance inflation factor (VIF), ranging from 1 to 1.27, suggested that there was no concern over collinearity. Unlike multiple linear regression, logistic regression does not assume normality or homoscedasticity. Overall, assumptions of logistic regression were met. Complete case analysis (i.e., listwise deletion) was used to address data with missing values. A two-sided *p* < 0.05 was considered statistically significant. Statistical analysis was conducted with SPSS version 26.

## 3. Results

### 3.1. Anxiety, Depression, and Health Behaviors among College-Student Clients

The demographic characteristics of 9884 participants are presented in Table 1. Results from the descriptive analysis showed that the mean (SD) GAD−7 score was 10.6 (5.8) in the study sample. The prevalence of moderate levels of anxiety was 53.5% based on the GAD−7 cutoff of 10, and the prevalence of severe levels of anxiety was 27.9% based on the cutoff of 15. Results from the descriptive analysis showed that the mean (SD) PHQ−9 score was 12.0 (6.7) in the study sample. The prevalence of moderate levels of depression was 58.2% based on the PHQ−9 cutoff of 10, and the prevalence of severe levels of depression was 34.6% based on the cutoff of 15.

Among participants who contracted COVID-19 during the pandemic period evaluated in this study (2020–2021), 56.8% experienced moderate levels of anxiety, and 30.2% experienced severe levels of anxiety. Further, 62.0% experienced moderate levels of depression, and 38.0% experienced severe levels of depression.

Table 1 shows a summary of college-student clients’ health behaviors. Approximately 90.8% of participants reported “All the time” when it was required to wear a facemask in public, 52.5% reported “Very closely” in terms of following recommendations for social/physical distancing, and 58.9% reported “Very closely” in terms of following recommendations for hygiene practices.

### 3.2. Main Effects

Table 2 shows a summary of stratified logistic regression models. The results revealed that adherence to masking, social distancing, and hygiene practices were significantly predictive of COVID-19 infection. In models controlling for anxiety symptom severity and covariates, better adherence to masking (aOR: 0.64, 95% CI: 0.58–0.70, *p* < 0.001), social distancing (aOR: 0.68, 95% CI: 0.63–0.72, *p* < 0.001), and hygiene practice (aOR: 0.82, 95% CI: 0.75–0.88, *p* < 0.001) predicted lower odds of COVID-19 infection. A one-unit increase in adherence to masking, social distancing, and hygiene practice was associated with 0.36, 0.32, and 0.18 lower odds of COVID-19 infection, respectively.

In models controlling for depressive symptom severity and covariates, better adherence to masking (aOR: 0.63, 95% CI: 0.57–0.69, *p* < 0.001), social distancing (aOR: 0.67, 95% CI: 0.63–0.72, *p* < 0.001), and hygiene practice (aOR: 0.84, 95% CI: 0.78–0.91, *p* < 0.001) predicted lower odds of COVID-19 infection. A one-unit increase in adherence to masking, social distancing, and hygiene practice was associated with 0.37, 0.33, and 0.16 lower odds of COVID-19 infection, respectively.

### 3.3. Interaction Effects of Anxiety/Depressive Symptom Severity

Table 3 shows a summary of stratified models with interaction terms. Regarding our first hypothesis, results revealed a significant interaction effect of anxiety symptom severity with social distancing (*b* = 0.016, *SE* = 0.006, *p* = 0.005) but not with masking or hygiene practice. As the severity of anxiety symptoms increased, the association of social distancing with COVID-19 infection became less negative.

Regarding our second hypothesis, results showed that the interaction effects of depressive symptom severity with masking (*b* = 0.020, *SE* = 0.007, *p* = 0.002), social distancing (*b* = 0.018, *SE* = 0.005, *p* < 0.001), and hygiene practice (*b* = 0.014, *SE* = 0.006, *p* = 0.02) were all statistically significant. As the severity of depressive symptoms increased, the association of masking, social distancing, or hygiene practices with COVID-19 infection became less negative.

## 4. Discussion

To our knowledge, this is one of the first studies to examine the potential moderating role of anxiety and depressive symptom severity in the protective effects of health behaviors against COVID-19 among college-student clients using mental health services. The results revealed a greater prevalence of moderate/severe anxiety [56.8%/30.2%] and depression [62.0%/38.0%] among clients who contracted COVID-19 than among all participants [53.5%/27.9% and 58.2%/34.6%], suggesting an increased vulnerability to COVID-19 infections among clients with these mental health conditions. The results of our models showing main effects (i.e., models without interactive terms) indicate that COVID-19 health behaviors provide clients with significant protection against COVID-19 when clients opt for better adherence, irrespective of the presence and severity of their anxiety or depressive symptoms. While the current literature has focused predominantly on the general population and healthcare workers [26,27], this research provides original insight into the extent of protective effects of these health behaviors against COVID-19 among college-student clients, who often experience varying levels of anxiety and depressive symptoms, supporting the protective effects of non-pharmaceutical prevention in mitigating the transmission of COVID-19. 

Further, the results showed that the severity of anxiety symptoms among college-student clients negatively moderated the protective effect of social distancing against COVID-19 infection, which supported our first hypothesis. This finding suggested that the protective effect of social distancing seemed less pronounced as clients experienced more severe anxiety symptoms, extending our current understanding of how anxiety symptom severity might undermine the protective effect of social distancing. One possible explanation is that severe anxiety symptoms can impair self-regulation and impulse control, leading to increased vulnerability to risky behaviors and heightened COVID-19 transmission risk [19,28]. College-student clients with more severe anxiety may disregard social distancing guidelines due to compromised decision-making, particularly in overcrowded settings where poor ventilation can further elevate infection risks [19,28,29,30]. The severity of anxiety might also affect the perception of social distancing adherence, with factors like rejection or isolation exacerbating risks by impairing judgment on attending social events, which could potentially increase COVID-19 exposure [31,32]. 

Additionally, the results revealed that college-student clients’ severity of depressive symptoms negatively moderated the protective effects of masking, social distancing, and hygiene practices against COVID-19, which supported our second hypothesis. This finding suggested that the greater severity of depressive symptoms experienced by the clients, the lesser the protective effects of clients’ health behaviors against COVID-19 infection. Previous research highlights how depressive symptoms detrimentally affect cognitive functions, including attention, memory, and perception, which are crucial for maintaining effective health behaviors [33,34]. These cognitive impairments, particularly in attentional control and executive function, are associated with difficulties in adhering to repetitive health tasks and making optimal decisions, such as engaging in preventive measures against COVID-19 [33]. This diminished cognitive capacity may result in riskier behaviors and hinder timely responses to health guidelines, like mask-wearing or maintaining a physical distance in over-crowded areas, further exacerbated by depressive symptoms’ impact on psychomotor speed, slowing physical and emotional reactions necessary for executing protective health behaviors promptly [35,36,37,38]. 

Lastly, socioeconomic challenges faced by clients with severe anxiety and depression might have impeded their ability to follow COVID-19 preventive measures due to factors like overcrowded living conditions, lower health literacy, and difficulty accessing reliable information and healthcare services [4,6,39]. These issues have called for targeted interventions, such as health education and accessible testing and vaccination programs, to support these college-student clients [4,6,40].

### 4.1. Limitations and Directions for Future Research

This study presents several limitations. First, the reliance on instruments collecting self-report data via a questionnaire and utilization of these retrospective, self-reported data introduces the potential for recall bias, thereby affecting the validity of the findings. The original survey included some instruments developed and standardized before the COVID-19 pandemic. While their application remains plausible during the pandemic era, the interpretation of the findings should be undertaken with caution. Further, results of this study may lack generalizability beyond the current college-student client population and study period. Among the 136,502 college students participating in the HMS survey, 126,618 were excluded from this study because they did not meet the inclusion criteria of this study; namely, they were not currently in mental health services as college-student clients. This said, future research may include both client and non-client populations to compare the potential moderating role of anxiety/depression in the protective effects of health behaviors. Considering some students of the non-client population also experiencing high levels of anxiety and depression, researchers can examine the potential differential protective effects of health behaviors associated with anxiety and depressive symptoms among this broader population. Moreover, it is noteworthy that the response rate for the Healthy Minds Study has seen a decline in recent years, registering at 15% for the current survey wave. While this decline poses the risk of non-response bias, it is important to recognize that sample weights were applied to mitigate this issue. Additionally, the institutions participating in the Healthy Minds Study varied annually. Although this variation in institutional characteristics between survey waves occurred randomly, prior research suggests that such variability is unlikely to significantly compromise the study’s results [24]. While we attempt to shed light on the potential mechanisms of these moderating effects of anxiety and depressive symptom severity on COVID-19 health behaviors through cognitive, behavioral, and socioeconomic lenses, limited literature has investigated this specific phenomenon. As a result, we were unable to compare our findings with other empirical evidence to elucidate the potential underlying mechanisms. This identifies a gap in the literature and underscores the need for further research to understand these mechanisms more comprehensively.

### 4.2. Implications for Health Policies and Clinical Practice

Implications emerging from this study’s findings may help inform health policies and clinical practices for better public health outcomes. First, the findings contribute to the growing body of evidence on the protective effects of non-pharmaceutical interventions in mitigating the transmission of COVID-19. Despite the distribution of COVID-19 vaccines—a pharmaceutical intervention—research has shown that their ability to prevent COVID-19 infection is limited due to the rapid emergence of more contagious variants [41,42,43]. Clients with mental health conditions are vulnerable to COVID-19 infection and severe clinical outcomes [4]. After infection, clients may not be able to receive timely antiviral treatment due to a lack of access to healthcare, shortages of antiviral medication, or exclusion criteria for prescription [44]. In these circumstances, clients can be at an increased risk for more severe COVID-19 outcomes. Thus, in addition to vaccines and medication, health behaviors should be considered critical public health tools in preventing worse COVID-19 outcomes across clients with varying levels of anxiety and depressive symptoms [41,45]. 

Second, findings from the present study suggest a potential public health benefit of clinicians’ efforts in assessing and addressing college students’ anxiety and depressive symptoms, namely, reducing clients’ vulnerability to COVID-19 infection, particularly during any surge of COVID-19 cases. As anxiety levels increase, clients may be more inclined to socialize even when such behaviors pose risks to their health. Physical distancing also requires individuals to stay aware of their surroundings to ensure an appropriate distance from others [46]. People with more severe depressive symptoms might inadvertently neglect to wear masks when transitioning from outdoor to indoor settings or neglect hand hygiene after contact with potentially contaminated surfaces. An inability to maintain attention to these health behaviors could lead to lapses or inconsistencies, inevitably exacerbating their risk of COVID-19 infection [29]. Clinicians (e.g., clinical psychologists, counselors, social workers, and psychiatrists) can apply motivational interviewing techniques to help clients process their thoughts and feelings and facilitate their behavior changes, such as clients’ health and mindfulness-related behaviors [47,48]. Mindful attention may assist clients in maintaining health behaviors while reducing their anxiety and depressive symptoms [48,49]. 

Taken together, clinicians are uniquely positioned to use appropriate psychological interventions and skills, helping clients address their mental health needs and the potential impact of mental health conditions on health behaviors, particularly those that pertain to public health and infectious disease prevention [47,48,50].

## 5. Conclusions

In this study, the severity of clients’ anxiety symptoms negatively moderated the protective effects of social distancing against COVID-19 infection. The severity of clients’ depressive symptoms negatively moderated the protective effects of masking, social distancing, and hygiene practices. The findings suggest the crucial role that mental health clinicians play in public health efforts aimed at mitigating the transmission of COVID-19 and perhaps other infectious diseases, especially among people with mental health conditions. The assessment and treatment of these mental health issues may contribute to collective public health initiatives between mental health clinicians and other health professionals in mental health promotion and infectious disease prevention.

## Figures and Tables

**Table 1:** Demographic Characteristics of the Sample, 2020–2021 (*n* = 9884).

Characteristic		Participants, No. (%) ^a^
Age		
	18–21	5161 (50.7)
	22–25	2184 (21.9)
	26–30	1229 (11.4)
	≥31	1310 (15.9)
Sex		
	Female	8278 (74.5)
	Male	1602 (25.4)
	Other	4 (0.1)
Race/ethnicity		
	American Indian/Alaskan Native	153 (2.2)
	Asian	611 (5.5)
	Black/African American	838 (9.2)
	Latinx	524 (5.1)
	Native Hawaiian/Pacific Islander	27 (0.3)
	Middle Eastern/Arab	81 (0.7)
	White	7544 (75.5)
	Other	106 (1.5)
Disability status		
	Yes	1888 (20.6)
	No	7996 (79.4)
Socioeconomic status (current financial stress)		
	Always stressful	1585 (17.9)
	Often stressful	2530 (26.8)
	Sometimes stressful	3353 (32.8)
	Rarely stressful	1849 (17.5)
	Never stressful	567 (5.1)
Geographic region		
	Northeast Region New England Division (Connecticut, Maine, Massachusetts, New Hampshire, Rhode Island, Vermont)	492 (3.5)
	Northeast Region Middle Atlantic Division (New Jersey, New York, Pennsylvania)	1570 (16.6)
	Midwest Region East North Central Division (Illinois, Indiana, Michigan, Ohio, Wisconsin)	2421 (21.9)
	Midwest Region West North Central Division (Iowa, Kansas, Minnesota, Missouri, Nebraska, North Dakota, South Dakota)	545 (6.6)
	South Region South Atlantic Division (Delaware, District of Columbia, Florida, Georgia, Maryland, North Carolina, South Carolina, Virginia, West Virginia)	2555 (21.4)
	South Region East South Central Division (Alabama, Kentucky, Mississippi, Tennessee)	538 (8.2)
	South Region West South Central Division (Arkansas, Louisiana, Oklahoma, Texas)	155 (2.8)
	West Region Mountain Division (Arizona, Colorado, Idaho, Montana, Nevada, New Mexico, Utah, Wyoming)	540 (6.8)
	West Region Pacific Division (Alaska, California, Hawaii, Oregon, Washington)	1068 (12.1)
Chronic health condition		
	Diabetes	164 (1.8)
	Hypertension	357 (5.1)
	Asthma	1778 (18.4)
	Thyroid disease	447 (4.8)
	Gastrointestinal disease	464 (4.3)
	Arthritis	321 (3.3)
	Sickle cell anemia	17 (0.1)
	Seizure disorders	117 (1.2)
	Cancers	93 (1.0)
	High cholesterol	338 (4.3)
	HIV/AIDS	12 (0.3)
	Other autoimmune disorder	347 (3.9)
	Other chronic disease	930 (9.2)
COVID-19 infection		
	Yes	2439 (25.6)
	No	7445 (74.4)
GAD−7		
	≥10 (moderate level of anxiety)	5267 (53.5)
	≥15 (severe level of anxiety)	2714 (27.9)
PHQ−9		
	≥10 (moderate level of depression)	5651 (58.2)
	≥15 (severe level of depression)	3291 (34.6)
Masking		
	All the time	9224 (90.8)
	Most of the time	544 (7.0)
	Sometimes	79 (1.2)
	Rarely	27 (0.7)
	Never	10 (0.3)
Social distancing		
	Very closely	5304 (52.5)
	Somewhat closely	3853 (39.5)
	Not closely	598 (6.5)
	Not at all	129 (1.6)
Hygiene practices		
	Very closely	5917 (58.9)
	Somewhat closely	3598 (36.9)
	Not closely	316 (3.6)
	Not at all	53 (0.5)

GAD−7: Generalized Anxiety Disorder–7; PHQ−9: Patient Health Questionnaire–9; ^a^ Percentages are weighted to be representative of the population at each institution.

**Table 2:** Main Effects of Health Behaviors on COVID-19 Infection in Stratified Models ^a,b^.

Variable	COVID-19 Infection	
	aOR (95% CI)	*p* Value
Model 1		
Anxiety symptom severity	1.02 (1.01–1.03)	<0.001
Masking	0.64 (0.58–0.70)	<0.001
Model 2		
Anxiety symptom severity	1.02 (1.01–1.03)	<0.001
Social distancing	0.68 (0.63–0.72)	<0.001
Model 3		
Anxiety symptom severity	1.02 (1.01–1.03)	<0.001
Hygiene practice	0.82 (0.75–0.88)	<0.001
Model 4		
Depressive symptom severity	1.02 (1.01–103)	<0.001
Masking	0.63 (0.57–0.69)	<0.001
Model 5		
Depressive symptom severity	1.02 (1.01–1.02)	<0.001
Social distancing	0.67 (0.63–0.72)	<0.001
Model 6		
Depressive symptom severity	1.02 (1.01–1.02)	<0.001
Hygiene practice	0.84 (0.78–0.91)	<0.001

aOR: adjusted odds ratio; CI: confidence interval; ^a^ adjusted for age, sex, race/ethnicity, disability status, socioeconomic status, geographic region, and chronic health conditions. ^b^ Predictors and covariates were entered into each stratified model at the same time.

**Table 3:** Interaction Effects of Anxiety and Depressive Symptom Severity with COVID-19 Health Behaviors ^a,b^.

Variable	COVID-19 Infection		
	Coefficient	Standard Error	*p* Value
Model 1			
Anxiety symptom severity × masking	0.012	0.008	0.11
Model 2			
Anxiety symptom severity × social distancing	**0.016**	0.006	0.005
Model 3			
Anxiety symptom severity × hygiene practice	0.002	0.007	0.72
Model 4			
Depressive symptom severity × masking	**0.020**	0.007	0.002
Model 5			
Depressive symptom severity × social distancing	**0.018**	0.005	<0.001
Model 6			
Depressive symptom severity × hygiene practice	**0.014**	0.006	0.02

Note: Bold font indicates statistical significance; ^a^ adjusted for age, biological sex, race/ethnicity, disability status, socioeconomic status, geographic region, and chronic health conditions; ^b^ in each stratified model, all variables were input at the same time.

## Data Availability

The data that support the findings of this study are openly available upon request in The Healthy Minds Network at https://healthymindsnetwork.org.
